# Identification of Schlafen-11 as a Target of CD47 Signaling That Regulates Sensitivity to Ionizing Radiation and Topoisomerase Inhibitors

**DOI:** 10.3389/fonc.2019.00994

**Published:** 2019-10-01

**Authors:** Sukhbir Kaur, Anthony L. Schwartz, David G. Jordan, David R. Soto-Pantoja, Bethany Kuo, Abdel G. Elkahloun, Lesley Mathews Griner, Craig J. Thomas, Marc Ferrer, Anish Thomas, Sai-Wen Tang, Vinodh N. Rajapakse, Yves Pommier, David D. Roberts

**Affiliations:** ^1^Laboratory of Pathology, Center for Cancer Research, National Cancer Institute, National Institutes of Health, Bethesda, MD, United States; ^2^Cancer Genetics Branch, National Human Genome Research Institute, National Institutes of Health, Bethesda, MD, United States; ^3^National Center for Advancing Translational Sciences, National Institutes of Health, Bethesda, MD, United States; ^4^Developmental Therapeutics Branch and Laboratory of Molecular Pharmacology, Center for Cancer Research, National Cancer Institute, National Institutes of Health, Bethesda, MD, United States

**Keywords:** radioresistance, epigenetics, CD47, thrombospondin-1, DNA damage response, schlafen-11, prostate cancer

## Abstract

Knockdown or gene disruption of the ubiquitously expressed cell surface receptor CD47 protects non-malignant cells from genotoxic stress caused by ionizing radiation or cytotoxic chemotherapy but sensitizes tumors in an immune competent host to genotoxic stress. The selective radioprotection of non-malignant cells is mediated in part by enhanced autophagy and protection of anabolic metabolism pathways, but differential H2AX activation kinetics suggested that the DNA damage response is also CD47-dependent. A high throughput screen of drug sensitivities indicated that CD47 expression selectively sensitizes Jurkat T cells to inhibitors of topoisomerases, which are known targets of Schlafen-11 (SLFN11). CD47 mRNA expression positively correlated with schlafen-11 mRNA expression in a subset of human cancers but not the corresponding non-malignant tissues. CD47 mRNA expression was also negatively correlated with *SLFN11* promoter methylation in some cancers. CD47 knockdown, gene disruption, or treatment with a CD47 function-blocking antibody decreased SLFN11 expression in Jurkat cells. The CD47 signaling ligand thrombospondin-1 also suppressed schlafen-11 expression in wild type but not CD47-deficient T cells. Re-expressing SLFN11 restored radiosensitivity to a CD47-deficient Jurkat cells. Disruption of CD47 in PC3 prostate cancer cells similarly decreased schlafen-11 expression and was associated with a CD47-dependent decrease in acetylation and increased methylation of histone H3 in the *SLFN11* promoter region. The ability of histone deacetylase or topoisomerase inhibitors to induce SLFN11 expression in PC3 cells was lost when *CD47* was targeted in these cells. Disrupting CD47 in PC3 cells increased resistance to etoposide but, in contrast to Jurkat cells, not to ionizing radiation. These data identify CD47 as a context-dependent regulator of *SLFN11* expression and suggest an approach to improve radiotherapy and chemotherapy responses by combining with CD47-targeted therapeutics.

## Introduction

CD47 is a widely expressed cell surface molecule in higher vertebrates (1, 2). CD47 plays a physiological role in recognition of self by serving as a counter-receptor for the inhibitory receptor SIRPα on macrophages and dendritic cells (3). CD47-like proteins acquired by *Poxviridae* also bind SIRPα and may have similar roles in protecting infected cells from host innate immunity (4, 5). Correspondingly, over-expression of CD47 in some cancers can protect tumors from innate immune surveillance (3, 6, 7). This has led to the development of therapeutic antibodies and decoy molecules that inhibit the CD47-SIRPα interaction and their entry into multiple clinical trials for cancer patients as potential innate immune checkpoint inhibitors (8–10).

In addition to the passive role of CD47 in self-recognition, cell-intrinsic signaling functions of CD47 have been identified in some tumor cells as well as in vascular and immune cells in the tumor microenvironment (11–13). CD47 signaling is induced by binding of its secreted ligand thrombospondin-1 (TSP1 encoded by *THBS1*), which modulates CD47 association with heterotrimeric G-proteins as well as lateral interactions of CD47 with specific integrins and tyrosine kinase receptors (1). In vascular cells, ligation of CD47 modulates calcium, nitric oxide, cAMP, and cGMP signaling (13). TSP1 also inhibits NK cell activation (14) and T cell receptor signaling in a CD47-dependent manner (15, 16). Genetic disruption or antisense suppression of CD47 enhances cytotoxic T cell killing of target tumor cells *in vitro* and suppresses tumor growth *in vivo* when combined with local tumor irradiation or cytotoxic chemotherapy (17, 18). In addition to enhancing their antitumor efficacy, blockade of CD47 signaling protects non-malignant tissues from the off-target effects of these genotoxic therapies by enhancing autophagy pathways, stem cell self-renewal, and broadly enhancing metabolic pathways to repair cell damage caused by ionizing radiation (19–21).

Here we utilized a high throughput screen of drug sensitivity to identify pathways that contribute to the radioresistance and chemoresistance of CD47-deficient cells. CD47-deficient cells exhibited significant resistance to topoisomerase and class I histone deacetylase (HDAC) inhibitors. Global differences in gene expression in WT Jurkat T cells and a CD47-deficient mutant and following siRNA knockdown of CD47 were examined to identify specific genes through which therapeutic targeting of CD47 could modulate radioresistance and chemoresistance. One of the genes that showed consistent down-regulation in CD47-deficient cells was *schlafen-11* (*SLFN11*), which in human cancers is positively correlated with sensitivity of cytotoxic agents including topoisomerase inhibitors (22–28). Loss of SLFN11 expression in cancer cells involves both hypermethylation of its promoter and epigenetic changes in histone modification (29, 30). Correspondingly, expression of *SLFN11* in some resistant cancer cell lines can be induced by class I HDAC inhibitors and restores their sensitivity, whereas knockdown of *SLFN11* confers resistance (29). The mechanism by which SLFN11 regulates sensitivity to DNA damaging agents includes limiting expression of the kinases ATM and ATR (31). Other evidence indicates that SLFN11 blocks DNA replication in stressed cells upon recruitment to the replication fork independent of ATR (32). Parallels between the effects of SLFN11 and CD47 on resistance to genotoxic stress suggested that SLFN11 may be an effector mediating the selective cytoprotective effects of CD47 knockdown, prompting us to examine the regulation of *SLFN11* and its orthologs by CD47 and the potential implications for combining CD47-targeted therapeutics with genotoxic cancer therapies.

## Materials and Methods

### Reagents and Cell Culture

Entinostat and rocilinostat were obtained from the NCI Division of Cancer Treatment and Diagnosis. Etoposide was from Bedford Laboratories. Doxorubicin was from Sigma-Aldrich.

PC3 and Jurkat T cells were purchased from the American Type Culture Collection and maintained at 37°C with 5% CO_2_ using RPMI 1640 medium supplemented with 10% FBS, glutamine, penicillin and streptomycin (Thermo Fisher Scientific, USA). The CD47-deficient Jurkat T cell mutant (clone JinB8) was from (33) and cultured as described previously (34). WT and CD47-deficient Jurkat cells were maintained at 2–5 × 10^5^ cells per ml to prevent activation.

For transient SLFN11 over-expression, 1 × 10^6^ JinB8 cells were transfected with 2 μg of SLFN11 expression vector (29) or control plasmid using an Amaxa nucleofection kit (Lonza) 48 h before irradiation. To assess cell viability Jurkat and JinB8 cells were plated at 2 × 10^4^ cells/well and irradiated with a single dose of 20 Gy radiation (operating at kV/10 mA with 2-mm aluminum filter, Precision X-Ray, East Haven, CT) or treated with etoposide. Cells were incubated for an additional 48–72 h at 37°C. Cell viability was determined by 3-(4,5-dimethylthiazol-2-yl)-5-(3-carboxymethoxyphenyl)-2-(4-sulfophenyl)-2H-tetrazolium, inner salt (MTS) reduction using the CellTiter 96 Aqueous One Solution proliferation assay (Promega). Absorbance was read at 490 nm on a microplate spectrophotometer.

PC3 cells and CD47-null CRISPR edited cells (2,000/well) were either unlabeled or labeled with Rapid Red Dye (Sartorius) and were plated in triplicate in 96-well plates (Corning, USA) and cultured overnight. The cells were treated as indicated in the figure legends, and cell proliferation was measured by Phase object Confluence (%) analysis using the IncuCyte instrument. Similarly, Jurkat and JinB8 T cells (5,000/well) were plated on 96 well plates for 1 h. The cells were treated with anti-human CD47 (B6H12, 1 μg/ml) as indicated in the figure legends.

400,000 WT and CD47 null PC3 cells were plated using 6 well plates in 2 ml of complete RPMI medium. The plates were irradiated with 20 Gy at a dose rate of 0.6 Gy/min using a GammaCell 40 Irradiator. PC3 cells were treated with entinostat, etoposide, doxorubicin, and rocilinostat at a concentration of 300 nM for 24 h for IncuCyte assays or for 72 h plated at 2,000 cells/well for MTS proliferation assays. Absorbance of untreated WT and CD47-null PC3 cells was normalized to 100%, and IC_50_ values were calculated using IC50 Calculator | AAT Bioquest software. Control and treated cells were also harvested for RNA extraction and real-time PCR.

### H2AX Assay

Jurkat and JinB8 cells or Jurkat cells pretreated with B6H12 antibody, were irradiated at 10 Gy, and then incubated for 0–6 h before fixing with paraformaldehyde for 15 min and washing 2 times with PBS. Cells were permeabilized using 0.14% Triton X-100 in PBS and 3% BSA for 5 min and washed three times for 5 min each. Then, cells were stained with H2AX primary antibody 1:300 for 60 min and secondary antibody 1:600 Alexa-fluor 488 (ebiosciences) for 60 min. Cells were washed three times with PBS and mounted using DAPI VECTASHIELD® Mounting Medium (Vector Laboratories, Inc, Burlingame, CA). Images were acquired using Zeiss 710 or Zeiss 780 microscopes with a 63x objective. High throughput antibody screening or quantification of FITC and DAPI was acquired on a Mirrorball instrument (TTP Labtech).

### Comet Assay

DNA fragmentation in WT and CD47^−^ Jurkat cells 24 h after irradiation at 10 Gy was assessed using a single cell gel electrophoresis (Comet) assay essentially as described (35).

### Real-Time PCR

Total RNA was extracted using the Trizol method (11, 34) or NucleoSpin RNA isolation kit (Clonetech), and the concentration and quality of RNA was measured using Nanodrop. First strand cDNA was generated using the Maxima First Strand cDNA Synthesis Kit for RT-qPCR, with dsDNase. All RNA samples were subjected to treatment with DNAse-1 prior to first strand cDNA synthesis according to the manufacturer's instructions (Thermo Fisher Scientific). Real-time PCR was performed using SYBR Green detection on a Bio-Rad CFX instrument as described previously (36).

For assessing responses to irradiation, total RNA was extracted as described above using TriPure isolation reagent (Roche). The concentration of RNA was measured using Nanodrop. Hundred nanogram and 1 μg of RNA was used to generate First strand cDNA using the Maxima First Strand cDNA Synthesis Kit for RT-qPCR, with dsDNase. mRNA expression of SLNF11 was amplified using SLFN11-F 5′-GGCCCAGACCAAGCCTTAAT-3′ and SLFN11-R, 5′-CACTGAAAGCCAGGGCAAAC-3′ primers with 1 μg of RNA template, while 18S and actin were amplified using 100 ng RNA. The relative expression is normalized to control untreated samples.

### Confocal Microscopy

PC3 cells (WT, low CD47, and CD47-null cells) were plated on 4-well ibidi chambers overnight. The next day, the cells were treated with Rocilinostat or Entinostat for 24 h. The cells were fixed with 4% paraformaldehyde (Sigma Aldrich), and immunostaining was performed using antibodies against CD47 (Proteintech Group, Inc) and SLFN11 (Santa Cruz Biotechnologies) as described previously (36). The images were captured using a Zeiss 710 microscope with an oil immersion 63x objective. Cells were treated with 300 nM of Rocilinostat, or Entinostat, or etoposide for 24 h, and the cells were immunostained using anti-SLFN11 as described above. The images were captured using a Zeiss 780 microscope with an oil immersion 63X objective. All the images were captured with 5 and 10 μm scale bars as indicated in the legends.

### CD47 Knockdown and Microarray Analysis

CD47 knockdown was performed using Jurkat T cells with CD47-siRNA as described earlier (34). Oligofectamine transfection reagent alone was used as mock control. Total RNA was extracted using the Trizol method. The quality of RNA was checked using a RNA Bioanalyzer (Agilent Inc). Global expression analysis was performed using Affymetrix microarray protocols as described previously (34, 36). Disruption of CD47 in PC3 cells was performed using a human CD47 gRNA targeting the first exon, 5′-CAGCAACAGCGCCGCTACCAGGG (37) using Cas9-GFP plasmid from Addgene (Cambridge, MA). The CRISPR plasmid was transfected using Oligofectamine (Thermo Fisher Scientific) according to the manufacturer's instructions. The cells were sorted based on CD47 expression to isolate CD47-low and CD47-null populations using CD47-PE antibody (Biolegend). The cells were expanded, and CD47 expression was re-validated by flow cytometry analysis using CD47-APC (Biolegend). CD47 Human siRNA Oligo Duplex (Locus ID 961) was purchased from OriGene and transfected using Oligofectamine (Thermo Fisher Scientific) into PC3 cells for 24 h using a 15 nanomolar concentration of the pooled CD47-siRNAs. After 24 h, the medium was removed, the cells were washed with PBS, and the cells were lysed using Tri Pure Isolation reagents. The knockdown of CD47-siRNA was assessed using real time PCR with the following primers: CD47-F (GGTTTGAGTATCTTAGCTCTAGCA), Long CD47-R (TCTACAGCTTTCCTAGGA) and short CD47-R (CCATCACTTCACTTCAGTCAGTTATTC).

### Human Tumor Expression Data

The Cancer Genome Atlas (TCGA) data was analyzed using cBioPortal tools to determine correlations between SLFN11 and CD47 mRNA expression in human tumors with sufficient RNAseq data (38, 39). Additional TCGA SLFN11 vs. CD47 mRNA expression plots and correlations were derived using log2(x + 1) transformed RSEM normalized count data obtained from the TCGA data portal (prostate adenocarcinoma and normal prostate tissue, invasive breast carcinoma and normal breast tissue, lung squamous cell carcinoma tissue). For some cancer types, correlations between SLFN11 or CD47 mRNA expression and *SLFN11* promoter DNA methylation were evaluated using TCGA methylation data derived using the Illumina HumanMethylation450 (HM450) BeadChip.

### Quantitative High-Throughput Drug Sensitivity Screen

Wild type Jurkat T cells and the CD47-deficient mutant JinB8 cells were seeded into 1,536-well plates at 500 cells per well, in 5 μL of medium. A library of FDA approved small molecule drugs and late preclinical stage compounds were added at multiple doses ranging from 0.8 nM to 46 μM. Cell viability was assessed after 48-h incubation at 37°C by adding 3 μL of CellTiter-Glo reagent (Promega) and measuring luminescence (RLU) after a 15 min incubation at 25°C, with ViewLux (PerkinElmer). Data from the high throughput screening assays was analyzed as previously described (18). Differential activity of each compound between the wild type and CD47 deficient cell lines was determined by calculating a difference in the maximum response or logIC_50_ (concentration giving 50% of maximal inhibition) for each compound.

### Chromatin Immunoprecipitation (ChIP)

WT CD47-low and CD47-null PC3 cells were plated overnight in 6-Well plates. The cells were fixed using Paraformaldehyde solution (Sigma), and chromatin was extracted using Chromatin Isolation kit (Abcam). Genomic DNA was sheared using 34 pulses of 10–12 s each at level by sonication with Disruptor sonication System from (Diagenode). ChIP was performed following instructions from ChIP Kit—One Step (Abcam). The Anti-Histone H3 (tri methyl K27) antibody, Anti-Histone H3 (di methyl K4) antibody and Anti-Histone H3 (acetyl K18) antibodies (Abcam) was incubated overnight for 4°C. The genomic SLFN11 primers were designed by using genomic DNA region of hg38_dna range = chr17:35373531-35374940 using UCSC Genome Browser. The following primers were designed using the Primer 3 program: SLFN-838 (CCGTCACGCTGCTAGTGATA), SLFN-968 (GAGTTGGCCAAAGACAGGAG), SLFN-949 (CTCCTGTCTTTGGCCAACTC), SLFN-1076 (CTCCGCATCAGTGAGAAGTG). SLFN11 level of eluted CHIP-DNA was measured using real-time genomic *SLFN11* primers with control GAPDH (Abcam). Enrichment in the ChIP assay was calculated by normalizing to the input.

### Statistical Analysis

Two-sample t-test assuming equal variances was used for cell viability assays to quantify statistical significance (^*^ for *p*-value < 0.05 and ^***^ for *p* < 0.001). Two-factor with replication ANOVA was used for real-time PCR analysis (^*^ for *p*-value < 0.05 and ^***^ for *p* < 0.001).

## Results

### CD47 Mutation or Antibody Engagement Modulates the DNA Damage Response

**Figure 1:**
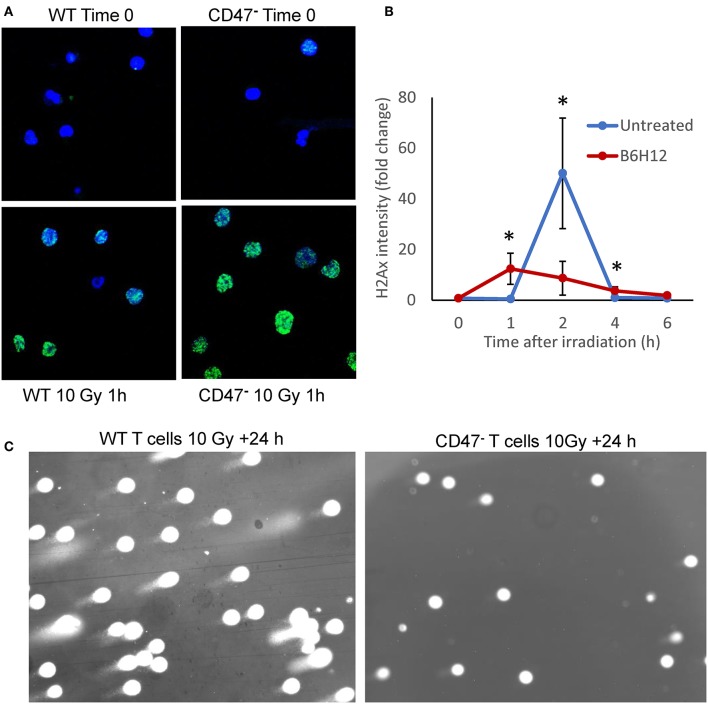
Mutation or blockade of CD47 improves the DNA damage response to ionizing radiation in Jurkat T cells. **(A)** Nuclear H2AX foci (green) stained in WT and CD47^−^ Jurkat T cells 1 h after irradiation at 10 Gy. **(B)** Modulation of the kinetics of H2AX foci formation by the CD47 antibody B6H12 in WT T cells after 10 Gy irradiation. **(C)** Comet Assay to detect DNA fragmentation in WT and CD47^−^ T cells 24 h after irradiation at 10 Gy. *p < 0.05.

We previously reported that non-malignant cells and Jurkat T cells lacking CD47 are protected from genotoxic stress induced by ionizing radiation (19, 21). This protection is mediated in part by an enhanced protective autophagy response in cells lacking CD47 or with reduced CD47 expression (19). Radioresistance in the CD47-deficient mutant is associated with global metabolic stabilization, including induction of anabolic metabolites that mediate repair of DNA damage induced by ionizing radiation (21). To evaluate whether CD47 also regulates the repair of genomic DNA damage caused by ionizing radiation, we assessed nuclear H2AX foci in WT and CD47^−^ Jurkat T cells 1 h after irradiation at 10 Gy (Figure 1A). Notably, the CD47^−^ cells showed a stronger H2AX response at this time. Quantitative analysis of the kinetics of foci formation in WT cells showed a maximal response at 2 h and subsequent decline by 6 h (Figure 1B). Previous studies of WT Jurkat cells subjected to this dose of radiation demonstrated metabolic collapse at 8 h followed by cell death (21), but treating WT cells with the CD47 function-blocking antibody B6H12 protected cells from radiation-induced death (40). Consistent with these results, subjecting the WT T cells to 10 Gy irradiation in the presence of B6H12 resulted in accelerated but less intense H2AX foci formation that resolved by 6 h (Figure 1B). A comet assay to assess DNA fragmentation in the CD47^−^ T cells 24 h after irradiation at 10 Gy showed no detectable DNA fragments, whereas DNA fragmentation at 24 h remained extensive in the irradiated WT cells (Figure 1C). Therefore, loss of CD47 or blocking its function improves the ability of these cells to restore genomic integrity after damage caused by ionizing radiation.

### Lack of CD47 Protects T Cells From Topoisomerase and HDAC Inhibitors

**Figure 2:**
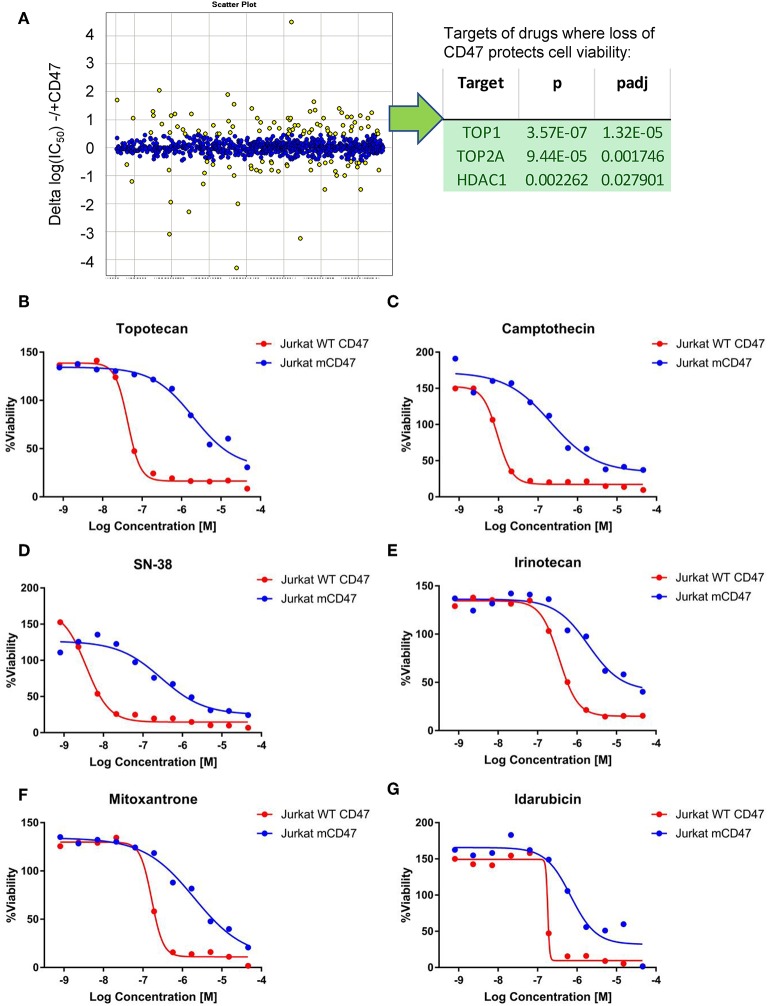
Loss of CD47 confers selective cytoprotection against cytotoxic drugs. **(A)** High throughput screen of FDA-approved and late stage development drugs. Cumulative data is presented as log(IC_50_) values comparing treated cells lacking or expressing CD47. Yellow points indicate compounds where the IC_50_ values differ significantly, and those with positive values indicate compounds where the absence of CD47 protects cell viability. The table lists the identified significant target classes for 72 compounds that exhibited >3-fold decreased potency (ΔIC_50_ for compounds with Curve Response Class −1.n and −2.n) in CD47-deficient Jurkat T cells compared to WT cells. **(B–G)** Representative dose response curves for WT Jurkat T cells (red) and CD47- mutant cells (blue). CellTiter Glo signal assessing cellular ATP levels is plotted as a function of Log(concentration) for topotecan **(B)**, camptothecin **(C)**, SN38 **(D)**, Irinotecan **(E)**, mitoxantrone **(F)**, and Idarubicin **(G)**.

Non-malignant cells lacking CD47 are also protected from genotoxic stress induced by the anthracycline doxorubicin (18), which causes DNA damage by multiple mechanisms including redox stress and inhibition of topoisomerase activity (41). A quantitative high-throughput screen of drug sensitivity was performed using the WT and CD47^−^ Jurkat T cell lines to identify additional drugs that may exhibit CD47-dependent cytotoxic activities and the resistance pathways they target. In addition to increased resistance to anthracyclines in the CD47-deficient cells, analysis of the 72 drugs that exhibited significantly decreased potencies (>3-fold) in CD47-deficient cells identified significant enrichments of topoisomerase I (TOP1), topoisomerase II (TOP2), and HDAC1 inhibitors (Figure 2A). Topoisomerase I inhibitors exhibited 5- to 100-fold increases in their IC_50_ values in the CD47-deficient cells (Figures 2B–E, Supplementary Data File 1). These included camptothecin, its therapeutic analogs topotecan and irinotecan, and the highly active irinotecan metabolite SN-38. Resistance of the CD47^−^ T cells extended to several drugs in the anthracycline family including idarubicin and mitoxantrone (Figures 2F,G).

**Figure 3:**
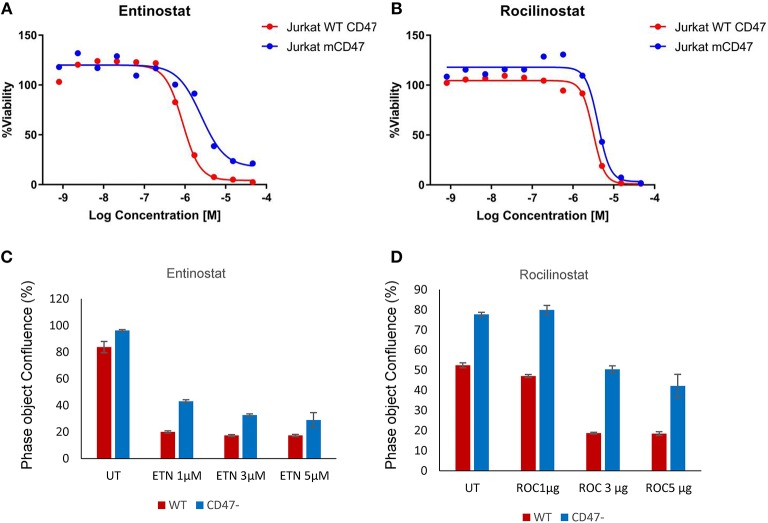
Loss of CD47 in Jurkat T cells increases resistance to selective HDAC1 and HDAC6 inhibitors. **(A,B)** WT (red) and CD47^−^ Jurkat T cells (blue) were treated with the indicated concentrations of Entinostat (HDAC1 selective) or Rocilinostat (HDAC6 selective), and cellular ATP was assessed using the CellTiter Glo reagent. **(C,D)** WT and CD47^−^ Jurkat T cells were treated with the indicated concentrations of Entinostat or Rocilinostat, and cell proliferation after 3 days was assessed by object counting using the IncuCyte live cell analysis instrument.

CD47^−^ cells exhibited enhanced resistance to 16 HDAC1 inhibitors in the screen (Supplementary Data File 1). One of these, the class I HDAC inhibitor entinostat (HDAC1>HDAC3), was previously shown to restore SLFN11 expression and sensitivity to DNA damage in resistant cancer cell lines (29). The IC_50_ value for entinostat was 3.2-fold higher for the CD47^−^ cells compared to WT cells in the CellTiter Glo assay (Figure 3A) and was confirmed to be less potent for inhibiting proliferation of CD47^−^ cells (Figure 3C). However, the class I HDAC inhibitor Romidepsin, which was shown to similarly induce SLFN11 (29), did not show differential activity in CD47^−^ vs. WT cells (Supplementary Data File 1). Conversely, the selective HDAC6 inhibitor Rocilinostat, which did not induce SLFN11 in K562 chronic myelogenous leukemia or HT1080 fibrosarcoma cells (29), was 1.51-fold less potent for CD47^−^ vs. WT cells in the CellTiter Glo assay (Figure 3B) and was less potent for inhibiting proliferation of CD47^−^ cells (Figure 3D). Therefore, some but not all of the differences in drug sensitivities between WT and CD47^−^ Jurkat cells are consistent with the previously reported effects of these drugs on SLFN11 expression.

### CD47 Correlation With *SLFN11* Gene Expression

**Figure 4:**
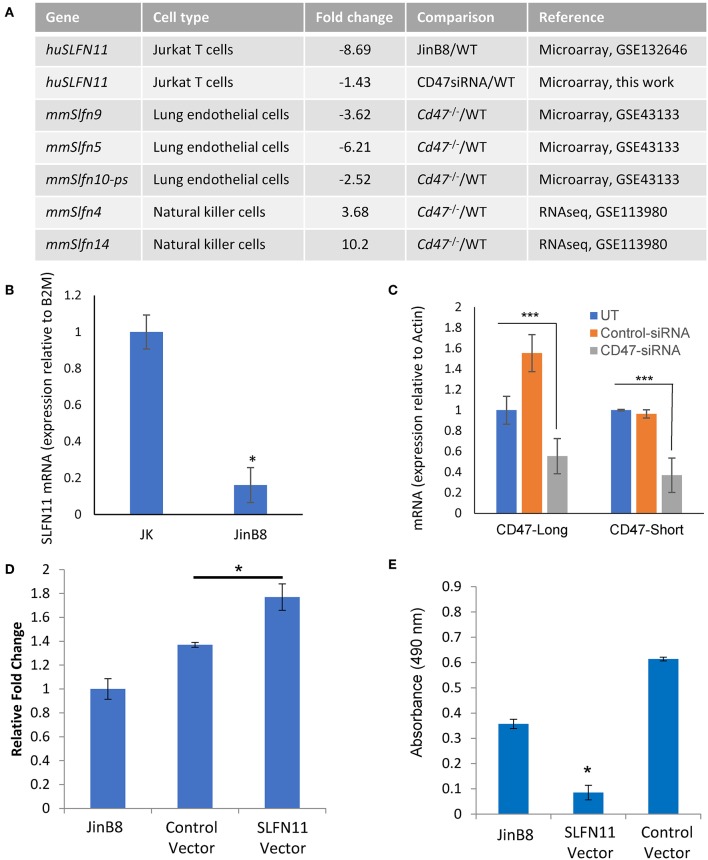
CD47-dependent *Schlafen* gene expression in human and murine cells. **(A)** Genes listed from the cited microarray and RNAseq studies scored *P*-value < 0.05. **(B)** RT-qPCR validation of reduced SLFN11 mRNA expression in WT and CD47^−^ JinB8 Jurkat T cells. **(C)** RT-qPCR confirmation of siRNA knockdown of the long and short CD47 mRNA transcripts. **(D)** RT-qPCR confirmation of transient over-expression of SLFN11 by plasmid transfection. **(E)** Radioresistance was assessed by MTS assay after irradiation at 20 Gy of CD47^−^ JinB8 cells transfected as indicated in **(D)**. *p < 0.05, ***p < 0.001.

Two independent microarray analyses of the same WT and CD47-deficient T cell lines identified 8.7- and 10-fold decreased expression of SLFN11 mRNA in the CD47-deficient Jurkat mutant (p < 0.05, Figure 4A). No other *Schlafen* gene family members showed a significant difference in expression between WT and CD47-deficient cells using a 2-fold cutoff. Decreased SLFN11 mRNA in the CD47^−^ mutant was confirmed using real-time qPCR (Figure 4B).

Reexamination of our published microarray data comparing primary lung endothelial cells from WT and *cd47*^−/−^ mice [GSE43133, (20)] identified a 3.6-fold decrease in mRNA expression of *Slfn9*, a presumed murine ortholog of the *SLFN11* gene (42), in *cd47*^−/−^ cells, suggesting that CD47 regulation of mRNA expression for *SLFN11* orthologs is conserved across species (Figure 4A). However, RNAseq analysis of unstimulated mouse NK cells (GSE113980) revealed increased *Slfn4* and *Slfn14* in *cd47*^−/−^ NK cells but no significant difference in *Slfn9* mRNA expression comparing sorted Lin^−^NK1.1^+^NKp46^+^ cells isolated from naïve *cd47*^−/−^ and *cd47*^+/+^ mouse spleens (43) (Figure 4A), suggesting that CD47 regulation of murine *Slfn9* expression is cell type-specific.

### CD47 Positively Regulates SLFN11 Expression

The decreased expression of SLFN11 in the CD47^−^ mutant T cells suggested that CD47 signaling regulates the expression of SLFN11. To establish causality and exclude the potential for secondary mutations in the Jurkat somatic mutant suppressing SLFN11 expression, we examined whether acute CD47 knockdown decreases schalfen-11 expression (Figures 4A,C, Supplementary Data File 2). siRNA knockdown of CD47 in the WT cells resulted in a 1.4-fold decrease in SLFN11 (*p* = 0.046). If loss of SLFN11 contributes to the radioresistance of the CD47^−^ cells, forced expression of SLFN11 should restore sensitivity. Transient transfection of the CD47^−^ cells with a SLFN11 expression vector increased SLFN mRNA expression (Figure 4D) and decreased the viability of CD47^−^ cells subjected to 20 Gy irradiation relative to untreated cells or cells transfected with the control plasmid (Figure 4E).

### CD47 Ligands Alter SLFN11 Expression

**Figure 5:**
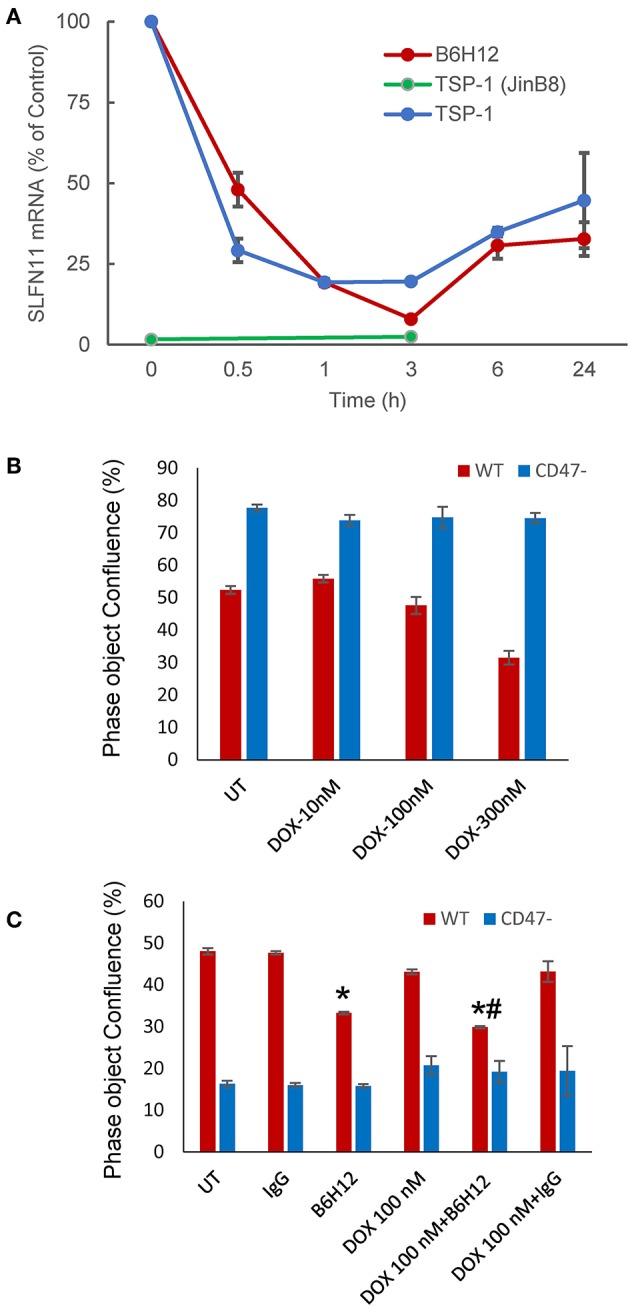
Modulation of SLFN11 expression and cell resistance by CD47 ligands. **(A)** TSP1 and CD47 antibody B6H12 inhibit SLFN11 expression. **(B)** Resistance of CD47-deficient Jurkat cells to the anti-proliferative activities of doxorubicin. Proliferation was assessed after 3 days by object confluence on the IncuCyte. **(C)** The CD47 antibody B6H12 increases sensitivity to doxorubicin. Proliferation was assessed after 4.5 days by object confluence on the IncuCyte live cell analysis system. Significance: * = vs. untreated, # = vs. B6H12 alone.

TSP1 signaling in T cells can be mediated by several cell surface receptors (44, 45), but at concentrations < 5 nM signaling is primarily CD47-dependent (15, 16). Correspondingly, treatment of WT but not CD47-deficient Jurkat T cells with 2.2 nM TSP1 time-dependently suppressed *SLFN11* mRNA expression (Figure 5A). Treatment of WT Jurkat T cells with TSP1 decreased SLFN11 mRNA as early as 30 min. after addition. Inhibition was maximal by 1–3 h and decreased thereafter. This time-dependence is consistent with the known uptake and degradation of TSP1 by T cells (45). No inhibition of SLFN11 mRNA expression by TSP1 was observed in the CD47^−^ mutant, indicating that the inhibitory effect of TSP1 on SLFN11 expression is CD47-dependent.

The function modifying CD47 antibody B6H12 has been used extensively as an antagonist of CD47 signaling through SIRPα in preclinical studies and demonstrated tumor suppressing activities in many xenograft models (3). However, some effects of B6H12 on cancer cells may be independent of inhibiting binding of CD47 ligands (11). B6H12 at 1 μg/ml rapidly suppressed SLFN11 mRNA expression in Jurkat cells, with variable recovery at later time points in repeated experiments (Figure 5A). This is consistent with the attenuated DNA damage response following irradiation of Jurkat cells in the presence of B6H12 in Figure 1B and the known ability of B6H12 treatment to preserve proliferative capacity in irradiated Jurkat cells (40).

A proliferation assay was used to examine whether the suppression of SLFN11 by B6H12 also alters the sensitivity of Jurkat cells to doxorubicin. A concentration of doxorubicin was selected that is suboptimal for directly inhibiting growth of the cells (Figures 5B,C). B6H12 alone at 1 μg/ml significantly inhibited cell proliferation, whereas an isotype-matched control antibody was inactive. Combining B6H12 with 100 nM doxorubicin resulted in more inhibition of cell growth, consistent with an additive effect (*p* = 0.001, Figure 5C). Therefore, this CD47 antibody can protect Jurkat cells from genotoxic stress induced by ionizing radiation or cytotoxic chemotherapy.

### Correlation Between CD47 and SLFN11 Expression in Human Cancers

An initial survey of TCGA cancer datasets with sufficient RNAseq data indicated that the positive correlation between CD47 and SLFN11 mRNA expression observed in Jurkat T cells extends to a subset of human cancers (Table 1). The most significant positive correlations were found for bladder urothelial carcinoma (*r* = 0.44, *p* = 8.75 × 10^−21^) and lung squamous cell carcinoma (*r* = 0.41, *p* = 2.2 × 10^−21^, Supplementary Figure 1A). Additional cancers with significant correlation included pediatric acute lymphoid leukemia, cutaneous melanoma, prostate adenocarcinoma, glioblastoma multiforme, hepatocellular carcinoma, esophageal carcinoma, invasive breast carcinoma, acute myeloid leukemia, soft tissue sarcoma, and renal clear cell carcinoma. The strong positive Spearman's correlation for lung squamous carcinoma contrasted with a lack of correlation for lung adenocarcinoma (*r* = −0.01, *p* = 0.89), further suggesting that the relationship between CD47 and SLFN11 expression is cancer type specific. Eight cancer types with adequate RNAseq expression data showed no significant correlation, and a significant negative correlation between CD47 and SLFN11 mRNA was observed for papillary thyroid carcinoma (*r* = −0.22, *p* = 3.4 × 10^−7^).

**Table 1:** Co-expression of SLFN11 and CD47 mRNA in human cancers.

**SLFN11/CD47**	**Spearman's Correlation**	***p*-Value**	***q*-Value**	***n***
Pediatric acute lymphoid leukemia	0.447	2.37 × 10^−11^	**1.19** **×** **10**^**−9**^	203
Bladder urothelial carcinoma	0.440	8.75 × 10^−21^	**2.21** **×** **10**^**−19**^	408
Lung squamous	0.407	2.25 × 10^−21^	**1.14** **×** **10**^**−19**^	501
Renal papillary cell carcinoma	0.355	4.51 × 10^−10^	**1.07** **×** **10**^**−8**^	291
Cutaneous melanoma	0.338	4.35 × 10^−14^	**6.44** **×** **10**^**−13**^	472
Prostate adenocarcinoma	0.329	5.01 × 10^−14^	**1.28** **×** **10**^**−12**^	498
Glioblastoma multiforme	0.269	4.52 × 10^−4^	**2.37** **×** **10**^**−3**^	166
Hepatocellular carcinoma	0.257	4.82 × 10^−7^	**6.91** **×** **10**^**−6**^	373
Esophageal carcinoma	0.256	4.28 × 10^−4^	**3.34** **×** **10**^**−3**^	185
Invasive breast carcinoma	0.245	1.70 × 10^−16^	**1.59** **×** **10**^**−15**^	1100
Acute myeloid leukemia	0.232	2.10 × 10^−3^	**6.45** **×** **10**^**−3**^	173
Soft tissue sarcoma	0.215	4.37 × 10^−4^	**2.17** **×** **10**^**−3**^	263
Renal CCC	0.169	8.88 × 10^−5^	**4.05** **×** **10**^**−4**^	534
Uterine endometrial carcinoma	0.165	0.0280	0.086	177
Pancreatic adenocarcinoma	0.160	0.0324	0.138	179
Brain lower grade glioma	0.086	0.048	0.072	530
Head and neck SCC	0.072	0.102	0.151	522
Colorectal adenocarcinoma	0.046	0.371	0.615	382
Lung adenocarcinoma	−0.006	0.892	0.926	517
Ovarian serous	−0.094	0.0985	0.234	307
Pediatric neuroblastoma	−0.097	0.251	0.445	143
Papillary thyroid carcinoma	−0.224	3.43 × 10^−7^	**1.01** **×** **10**^**−6**^	509

*RNAseq data from the indicated TCGA provisional datasets (except TARGET datasets for pediatric ALL and pediatric neuroblastoma) were analyzed using cBioPortal tools. q-values were derived from the Benjamini-Hochberg FDR correction procedure. Significant q-values are indicated in bold font*.

**Figure 6:**
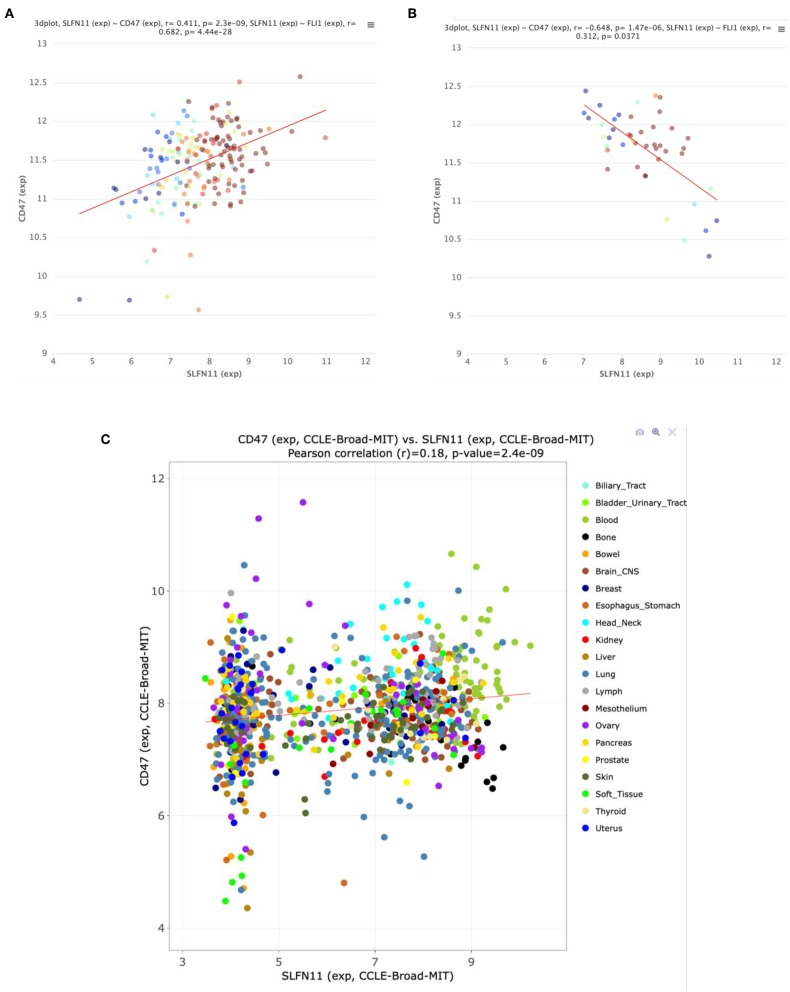
Correlation between CD47 and SLFN11 mRNA expression determined by RNAseq analysis of prostate cancers **(A)** and normal prostate tissues **(B)** in TCGA. Point colors indicate expression of FLI1, a known driver of *SLFN11* expression (26). **(C)** Scatter plot of CD47 and SLFN11 mRNA expression in the Cancer Cell Line Encyclopedia cell lines. Colors identify the indicated cancer origins. The data is publicly available at http://discover.nci.nih.gov/cellminercdb.

Further analysis of the TCGA data for prostate cancers showed a positive correlation between CD47 and SLFN11 mRNA expression in the tumors but not in normal prostate tissues (Figures 6A,B). Normal breast tissue similarly lacked the positive correlation between CD47 and SLFN11 observed in invasive breast carcinomas (Supplementary Figures 1B,C). These data further indicate that CD47 regulation of SLFN11 mRNA expression is cell type-specific and differs between normal and malignant tissues.

A positive correlation between CD47 and SLFN11 was also found for the cell lines in the Cancer Cell Line Encyclopedia (Spearman's correlation 0.193, *p* = 1.6 × 10^−9^, *q* = 2.6 × 10^−8^, Figure 6C). These data suggest that the underlying mechanism for these positive correlations is at least partially intrinsic to the cancer cells. SLFN11 mRNA expression in the CCLE was bimodal. Segregating high vs. low expressing cell lines with a mean cutoff showed higher CD47 in the SLFN11 high cell lines (log ratio 0.35, *p* = 3.1 × 10^−8^). Of the 7 prostate cancer cell lines in the CCLE, LNCAP and 22RV1 were high SLFN11 expressers, PC3 was moderate, and the remaining 4 were low expressers.

### Loss of CD47 Regulates SLFN11 Expression in Prostate Cancer Cells

**Figure 7:**
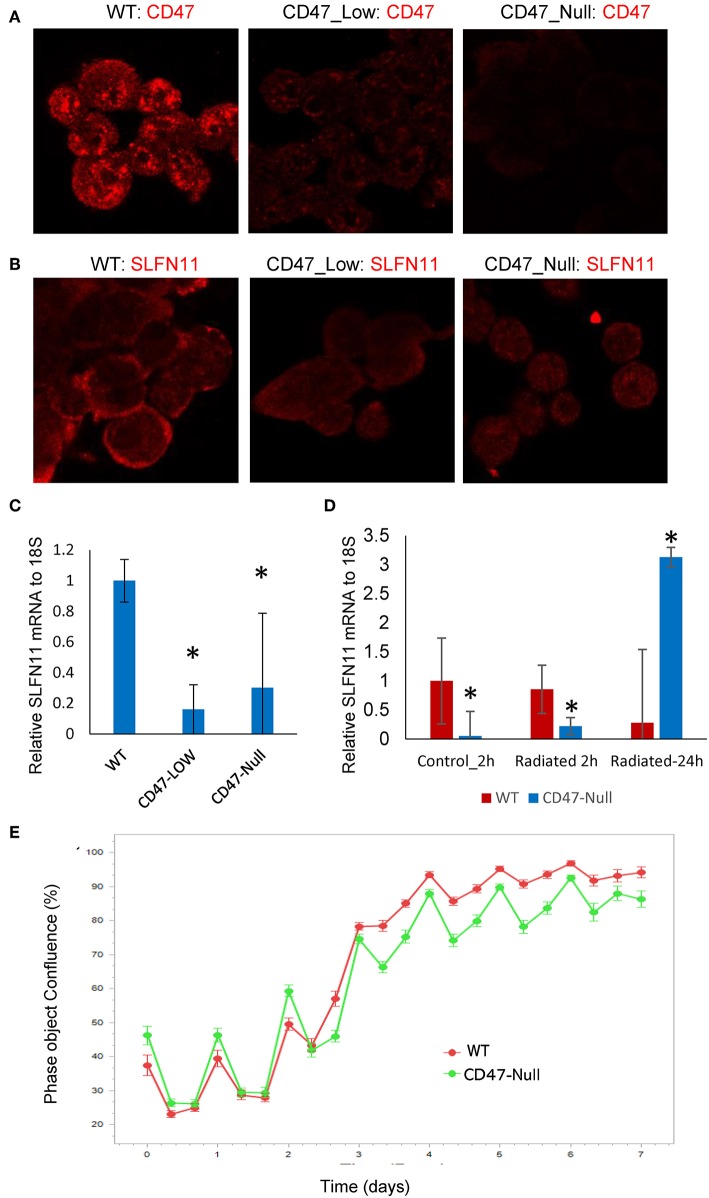
Generation and characterization of CD47-deficient PC3 prostate cancer cells. **(A)** Analysis of CD47 expression in CD47-CRISPR-targeted PC3 cells sorted based on low or absent cell surface CD47 expression. **(B)** SLFN11 expression in WT, Low-CD47, and CD47-null PC3 cells. **(C)** Quantitative analysis of data in **B**. **(D)** RT-qPCR analysis of SLFN11 mRNA expression in WT and CD47-null PC3 cells and the respective cells 2 or 24 h after irradiation at 20 Gy. **(E)** Proliferation of irradiated WT and CD47-null PC3 cells by object confluence on the IncuCyte live cell analysis system. *p < 0.05.

We chose the PC3 line to examine whether CD47 also regulates SLFN11 expression and sensitivity to genotoxic stress in prostate cancer cells. CD47 was targeted using CRISPR/Cas9, and pools of mutant PC3 cells with low residual CD47 or completely lacking CD47 were isolated by fluorescence activated cell sorting. Lack of or decreased CD47 expression was confirmed by flow cytometry and visualized by immunofluorescent staining (Figure 7A). The CD47-null PC3 cells proliferated at a somewhat slower rate that the WT PC3 cells (Supplementary Figure 2). Loss or absence of CD47 expression in PC3 cells was accompanied by decreased SLFN11protein expression (Figures 7B,C). SLFN11 mRNA expression was also reduced in the CD47-null PC3 cells (Figure 7D).

### Loss of CD47 Differentially Regulates Drug and Radiation Sensitivities in Prostate Cancer Cells

Although loss of CD47 in Jurkat cells consistently protects these cells from ionizing radiation [present results and (19, 21, 40)], this was not the case when CD47 was disrupted in PC3 cells (Figure 7E). The initial loss and recovery of WT and CD47-null PC3 cells after irradiation at 20 Gy were similar. SLFN11 mRNA remained lower 2 h post-irradiation in the CD47-null cells but rose above that in irradiated WT PC3 cells at 24 h (Figure 7D).

Consistent with their lower SLFN11 and with the Jurkat cell results, CD47-null PC3 cells were less sensitive to rocilinostat and etoposide than were the WT PC3 cells (Table 2, Supplementary Figure 3). In contrast, CD47-null PC3 cells were moderately more sensitive than WT cells to entinostat and doxorubicin.

**Table 2:** Drug sensitivities of WT and CD47-null PC3 cells.

**Drug**	**WT IC_**50**_ (μM)**	**CD47-null IC_**50**_ (μM)**
Etoposide	0.18	0.58
Doxorubicin	0.91	0.47
Rocilinostat	0.25	>0.30

**Figure 8:**
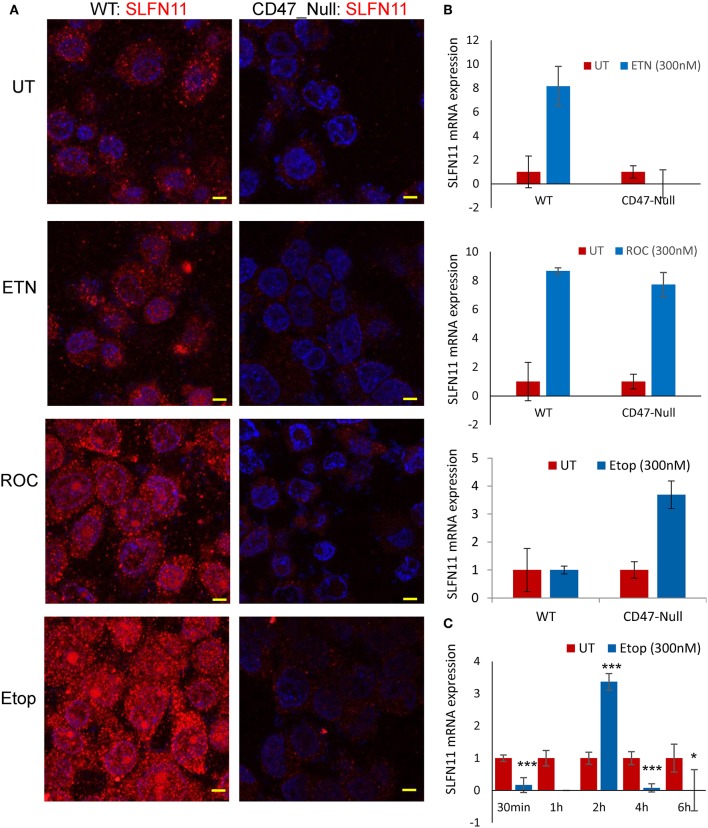
Stress induces SLFN11 expression in WT but not in CD47-null PC3 cells. **(A)** Immunofluorescent imaging of SLFN11 protein expression in untreated WT and CD47-null PC3 cells (UT), and cells treated 24 h with 300 nM entinostat (ETN), rocilinostat (ROC), or etoposide (Etop). **(B)** SLFN11 mRNA expression in cells following the respective treatments for 24 h. Expression was normalized to actin and to one for the untreated WT and CD47-null PC3 cells. **(C)** WT PC3 cells (~100,000/well) were plated overnight using complete RPM1 medium. After 24 h the cells were treated with 300 nM etoposide or an equal volume of PBS as control. The cells were harvested at the indicated time points, and 1 μg of total RNA per treatment was used to generate cDNA for RT-qPCR analysis. Relative SLFN11 mRNA expression was calculated using 18S RNA primer control and normalized to untreated controls for each time point (* for *p*-value < 0.05 and *** for *p* < 0.001).

Treating WT PC3 cells with a sublethal concentrations of rocilinostat, entinostat, or etoposide for 24 h increased SLFN11 protein levels as detected by immunofluorescence (Figure 8A). In contrast, SLFN11 expression in CD47-null PC3 cells was not significantly induced by the same treatments. Induction of SLFN11 protein by rocilinostat, entinostat was paralleled by increased SLFN11 mRNA at 24 h in WT PC3 cells (Figure 8B). No elevation in SLFN11 mRNA was observed at the same time point in WT PC3 cells treated with etoposide. However, a time course for treatment with 300 nM etoposide indicated acute induction of SLFN11 mRNA at 2 h following the initial decrease (Figure 8C), which may account for the elevation of SLFN11 protein seen at 24 h in Figure 8A. Notably, SLFN11 mRNA was up-regulated at 24 h in the CD47-null cells treated with rocilinostat or etoposide. These data suggest a CD47 context-dependent effect of HDAC inhibition on SLFN11 expression in PC3 cells.

### Tumor Type-Specific Correlation of CD47 Expression With *SLFN11* Promoter Methylation

**Figure 9:**
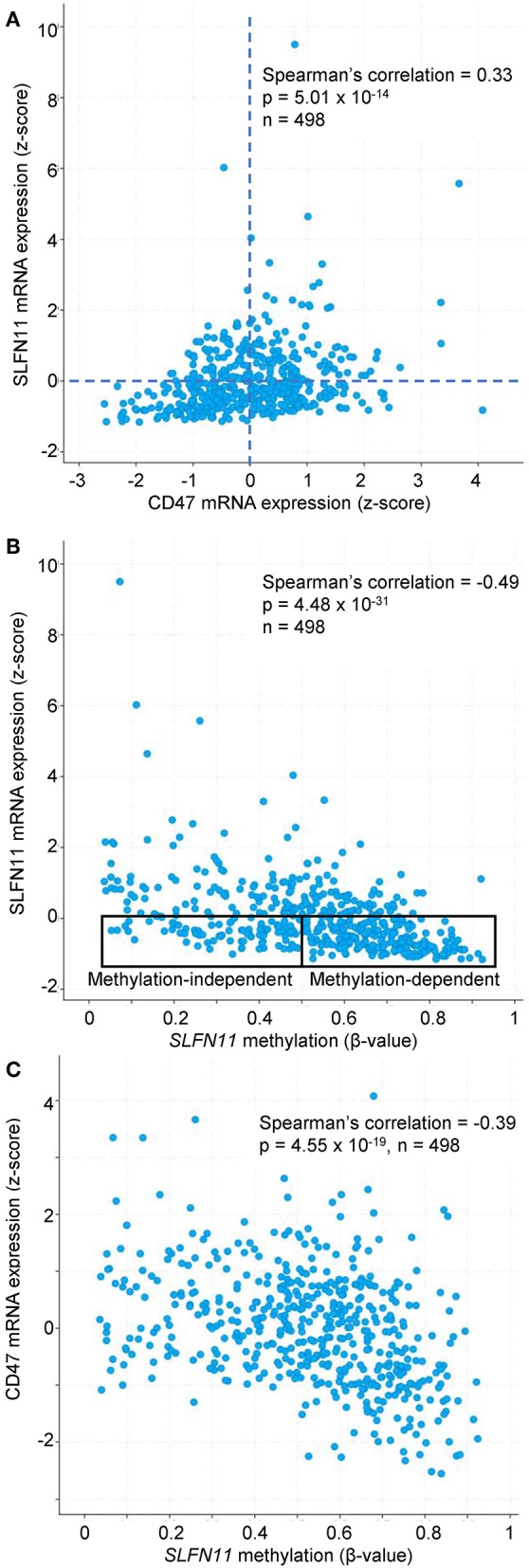
*SLFN11* promoter methylation in human prostate carcinomas correlates with SLFN11 and CD47 expression. TCGA data were analyzed using cBioPortal tools. **(A)** Positive correlation between z-scores for SLFN11 and CD47 mRNA expression determined by RNAseq analysis. **(B)** *z*-scores for SLFN11 mRNA expression are negatively correlated with β-values for *SLFN11* promoter methylation data from the Illumina HumanMethylation450 (HM450) BeadChip. The indicated subsets with low SLFN11 mRNA (*z* < 0) are predicted to be promoter methylation-dependent or -independent based on the previous *in vitro* analysis of tumor cell lines (29). **(C)** *SLFN11* promoter methylation is negatively correlated with CD47 mRNA expression.

Previous studies have identified roles for promoter methylation and epigenetic regulation in the loss of SLFN11 expression in various cancers (24, 29, 30). We further analyzed TCGA prostate cancer data to evaluate a potential role of CD47 in these two mechanisms for regulating *SLFN11* transcription. Consistent with the data in Figure 6A, prostate tumors with low SLFN11 mRNA (z-score < 0) were enriched in the quadrant with low CD47 mRNA (34% CD47 z-score < 0 vs. 26% *z*-score >0, Figure 9A). As reported previously for a broad collection of cancer cell lines (29), SLFN11 mRNA in prostate tumors was negatively correlated with methylation of the *SLFN11* promoter (*p* = 4.5 × 10^−31^, Figure 9B). A weaker negative correlation between CD47 mRNA expression and *SLFN11* promoter methylation (*p* = 4.6 × 10^−19^) suggested that the regulation of SLFN11 expression in human prostate cancers by CD47 is mediated in part by this mechanism (Figure 9C). However, another subset of the prostate cancers with low SLFN11 expression had low promoter methylation (Figure 9B), which was previously demonstrated using a diverse panel of cancer cell lines to predict epigenetic regulation of *SLFN11* (29).

TCGA data for additional cancer types in Table 1 were examined to determine the specificity of the correlation between CD47 expression and *SLFN11* promoter methylation. In all the cancer types where adequate methylation data was available, SLFN11 mRNA expression was negative correlated with *SLFN11* promoter methylation (Supplementary Figure 4, left panels). Consistent with the mRNA correlations in Table 1, CD47 mRNA expression in breast carcinomas was negatively correlated with *SLFN11* methylation (*p* = 3.6 × 10^−14^), and colorectal carcinoma lacked a significant correlation (*p* = 0.62). In contrast, the positive correlation between CD47 and SLFN11 expression for melanomas in Table 1 (*p* = 4.4 × 10^−14^) diverged from the weak negative correlation between CD47 expression and *SLFN11* methylation in these tumors (*p* = 3.1 × 10^−3^). Furthermore, soft tissue sarcomas exhibited low levels of *SLFN11* methylation that were independent of CD47 expression (*p* = 0.27), but had a significant positive correlation between CD47 and SLFN11 expression (*p* = 4.4 × 10^−4^), consistent with epigenetic cross-regulation independent of *SLFN11* promoter methylation in these tumors.

### Epigenetic Regulation of SLFN11 by CD47

**Figure 10:**
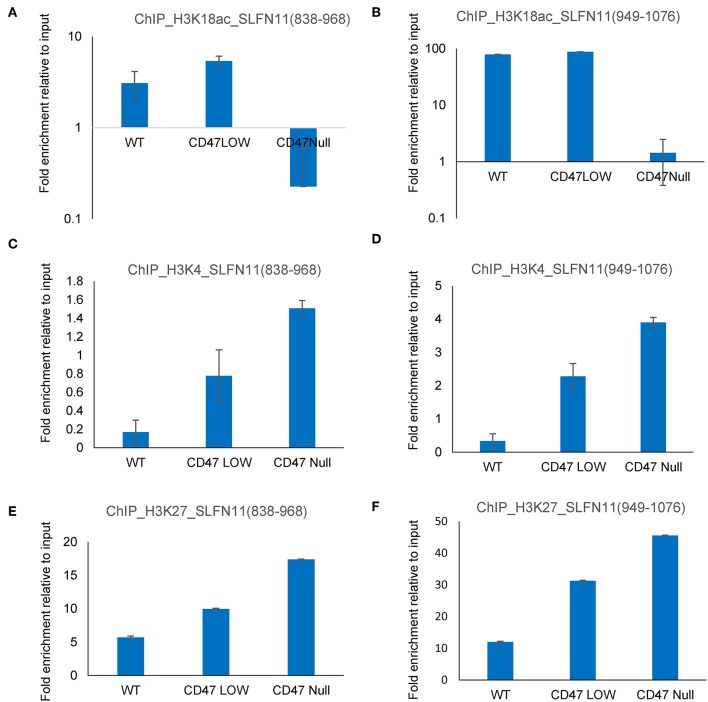
Epigenetic regulation of the *SLFN11* promoter in PC3 cells with altered CD47 expression. Chromosome immunoprecipitation from WT and CD47-null PC3 cells was performed using acetylated H3K18 **(A,B)**, trimethyl H3K4 **(C,D)**, and trimethylated H3K27 antibodies **(E,F)**. Immune precipitates were analyzed by PCR using primers to amplify 838–968 **(A,C,E)** or 949–1,076 bp **(B,D,F)** 5′ from hg38_dna range=chr17:35373531-35374940 5'pad in the *SLFN11* promoter. Results are expressed as fold enrichment relative to input.

The ability of selective HDAC1 inhibitors to restore SLFN11 expression in some resistant cancer cell lines (29) and the subset of prostate cancers in the TCGA data with low SLFN11 expression despite low promoter methylation suggested a potential epigenetic mechanism by which CD47 signaling could alter SLFN11 expression in prostate cancer. To examine potential epigenetic mechanisms for regulation of *SLFN11* gene expression by CD47 in prostate cancer cells, we performed chromosome immunoprecipitation in WT and CD47-null PC3 cells using acetylated H3K18, trimethylated H3K4, and trimethylated H3K27 antibodies and analyzed their enrichment in a region upstream from *SLFN11* that was identified based on ENCODE data to contain a high abundance of histone H3K27Me3 modification (Supplementary Figure 5). Consistent with the low *SLFN11* expression in the CD47-null PC3 cells, H3K18Ac enrichment was markedly decreased at 838–968 and 949–1,076 (Figures 10A,B). However, H3K18Ac enrichment did not show a corresponding decrease consistent with the decrease in SLFN11 mRNA expression in the CD47-low pool. In contrast, enrichment of trimethylated H3K4 and H3K27 was dose-dependent with decreasing CD47 expression (Figures 10C–F).

## Discussion

Previous studies have identified SLFN11 expression as a major determinant of cancer cell sensitivity to DNA-damaging chemotherapeutic agents and patient outcomes for several cancers (22–26, 29, 46). The present data extends this role to regulating the sensitivity of cells to ionizing radiation. We further identify a role for SLFN11 in the regulation by CD47 of the sensitivity of cells to radiotherapy and chemotherapy. Decreased expression of CD47 or its engagement by physiological or pharmacological ligands suppresses SLFN11 expression, and re-expression of SLFN11 in some cells with low CD47 expression is sufficient to restore their sensitivity to DNA damage. Previous studies have identified other cytoprotective pathways regulated by CD47 that are probably independent of SLFN11 including upregulation of autophagy (19, 47), anabolic metabolites (21), and transcription factors that support asymmetric stem cell self-renewal (20), but SLFN11 regulation by CD47 provides a complementary mechanism to more proximally regulate the DNA damage response.

Loss or blockade of CD47 in non-transformed cells and tissues and in the Jurkat T cell line consistently protects cells from genotoxic and ischemic stresses (1). However, some of the underlying protective mechanisms are lost or lead to different outcomes in cancer cells. For example, the protective autophagy response in non-transformed CD47-deficient cells exposed to ionizing radiation manifests as a non-protective mitophagy response in breast cancer cells (18). Similarly, blockade of CD47 signaling that preserves non-transformed stem cells results in differentiation of breast and hepatocellular carcinoma and stem cells (11, 12, 48). The present data demonstrate a similar divergence in regulation of the SLFN11 pathway in different cell lines. Loss of CD47 coincides with loss of expression for *SLFN11* or its presumed murine ortholog Slfn9 in non-transformed cells, and at least in human Jurkat cells this contributes to protection from genotoxic stress induced by ionizing radiation or cytotoxic chemotherapy. Transient over-expression of SLFN11 is sufficient to resensitize CD47-deficient Jurkat cells to ionizing radiation. Conversely, ligation of CD47 by a CD47 antibody, which is known to confer cytoprotection in WT Jurkat cells (40), rapidly decreases SLFN11 expression in these cells. The physiological CD47 signaling ligand TSP1 similarly induces a decrease in SLFN11 expression.

The positive regulation of *SLFN11* expression by CD47 extends to prostate cancer cells, and correlative data in human tumors extends this relationship to a subset of human cancers. Our PC3 cell data indicates that CD47 regulation of *SLFN11* and responses to stress is more restricted in this cancer cell line. The decreased SLFN11 in CD47-null PC3 cells was not sufficient to protect these cells from ionizing radiation, but resistance to the anti-proliferative effects of etoposide and rocilinostat were observed. The latter resistance is consistent with the inability of rocilinostat to induce SLFN11 protein expression in the CD47-null PC3 cells.

To interpret the differences in *SLFN11* regulation in WT and CD47-deficient cells following exposure to ionizing radiation, DNA damaging agents, or HDAC inhibitors, it is important to recognize the temporal differences in their action. DNA strand breaks are induced rapidly by radiation and locally produced ROS, and all damage occurs over a few minutes. In contrast, doxorubicin causes cumulative DNA damage by several mechanisms including intercalation, ROS-induced strand breaks, and inhibition of topoisomerase activity. Etoposide is a more specific inhibitor of topoisomerase activity, but this activity is similarly sustained for the duration of treatment. Our data show that sustained exposure to doxorubicin or etoposide results in a CD47-dependent accumulation of SLFN11 over 24 h. The HDAC inhibitors similarly induce SLFN11 in a CD47-dependent manner. Looking only at 24 h, radiation appears to induce the opposite response. SLFN11 mRNA was induced in CD47-null PC3 cells but not in irradiated WT cells. Considering the noted temporal differences in the respective genotoxic stresses, the observed CD47-dependence for *SLFN11* regulation by these stresses may be consistent.

The detailed molecular mechanism by which CD47 signaling regulates SLFN11 mRNA and protein levels remains to be determined. The high throughput drug screen identified a significant resistance of CD47^−^ Jurkat cells to class I HDAC inhibitors including the selective HDAC1 entinostat. On the other hand, lack of differential activity for romidepsin and differential inhibition of the CD47^−^ and WT cells by the selective HDAC6 inhibitor rocilinostat suggested that the regulation of SLFN11 by CD47 may not exclusively involve HDAC1. Consistent with the drug screening data, ChIP data identified CD47-dependent regulation of histone modification in the *SLFN11* promoter in prostate cancer cells. Loss of CD47 in PC3 cells was associated with increased histone H3 K4 methylation and K27 methylation and decreased H3K18 acetylation at this locus. The observed epigenetic effects of CD47 signaling on *SLFN11* could also account for the differential resistance of CD47-deficient Jurkat T cells to several HDAC inhibitors in the drug screening. However, the ability of etoposide and rocilinostat to induce SLFN11 mRNA without a corresponding increase in protein expression in CD47-null cells suggests that CD47 positively controls SLFN11 expression by a post-transcriptional mechanism.

Analysis of tumor data in TCGA also implicated CD47 regulation of *SLFN11* promoter methylation in a subset of cancers that includes prostate adenocarcinoma. However, *SLFN11* promoter methylation is independent of CD47 expression in some cancer types that exhibit a positive correlation between SLFN11 and CD47 mRNA expression. Further studies will be required to define the relative importance of these two mechanisms in the cross talk between CD47 and SLFN11 in each cancer type. These data could guide the design of clinical trials combining CD47-targeted therapeutics with anticancer drugs that target DNA methylation or histone modification to maximize therapeutic responses in each cancer.

## Data Availability Statement

All datasets generated for this study are included in the manuscript/Supplementary Files.

## Ethics Statement

Ethical review and approval was not required for the study on human participants in accordance with the local legislation and institutional requirements. The patients/participants provided their written informed consent to participate in this study.

## Author Contributions

SK, AS AT, S-WT, YP, and DR contributed to conception and design of the study. SK, AS, DJ, DS-P, BK, AE, LM, CT, MF, VR, YP, and DR performed acquisition, analysis, or interpretation of data. SK, AS, and AE performed the statistical analysis. SK, AS, and DR wrote the first draft of the manuscript. DS-P wrote sections of the manuscript. All authors contributed to manuscript revision, read, and approved the submitted version.

### Conflict of Interest

AS is Chief Executive Officer and a shareholder of Morphiex Biotherapeutics. The remaining authors declare that the research was conducted in the absence of any commercial or financial relationships that could be construed as a potential conflict of interest.
