# Supplementary material for: Identification of Schlafen-11 as a Target of CD47 Signaling That Regulates Sensitivity to Ionizing Radiation and Topoisomerase Inhibitors
Source: Front Oncol. 2019 Oct 1;9:994. doi: 10.3389/fonc.2019.00994 (PMC6781860; doi:10.3389/fonc.2019.00994)

Supplementary Material

1 Supplementary Data

Data file 1. Excel spreadsheet analyzing HTS drug screening data for WT and $CD47^-$ Jurkat T cells:

Supplementary data drug screen CD47 KO Jurkat.xlsx

Data file 2. Excel spreadsheet listing genes significantly altered following siRNA knockdown of CD47 in Jurkat T cells:

Supplementary data CD47 siRNA microarray analysis.xlsx

2 Supplementary Figures

2.1 Supplementary Figure Legends

Supplementary Figure 1. Correlation between CD47 and SLFN11 mRNA expression in TCGA lung squamous adenocarcinoma date (A). Point colors indicate expression of FLI1, a known driver of *SLFN11* expression (26). Positive correlation between SLFN11 and CD47 in TCGA invasive breast carcinoma samples (B) but not normal breast tissue (C).

Supplementary Figure 2. Proliferation of WT and CD47-null PC3 cells assessed by object counting on the Incucyte instrument.

Supplementary Figure 3. Inhibition of WT and CD47-null PC3 cell proliferation by entinostat (A) and rocilinostat (B) was assessed by object counting on the Incucyte instrument. Dose response for inhibition by doxorubicin (C) and etoposide (D) was assessed colorimetrically using the MTS assay and analyzed using https://www.aatbio.com/tools/ic50-calculator.

Supplementary Figure 4. *SLFN11* promoter methylation in human cancers and its correlation with CD47 expression. TCGA data for the indicated cancers were analyzed using cBioPortal tools. The left panels show z-scores for SLFN11 mRNA expression plotted versus β-values for *SLFN11* promoter methylation assessed using the Illumina HumanMethylation450 (HM450) BeadChip. The right panels show SLFN11 methylation data plotted versus CD47 mRNA expression determined by RNAseq.

Supplementary Figure 5. Epigenetic profile of the 5’ region of *SLFN11* based on ENCODE data for the indicated cell lines.

Supplementary Figure 1

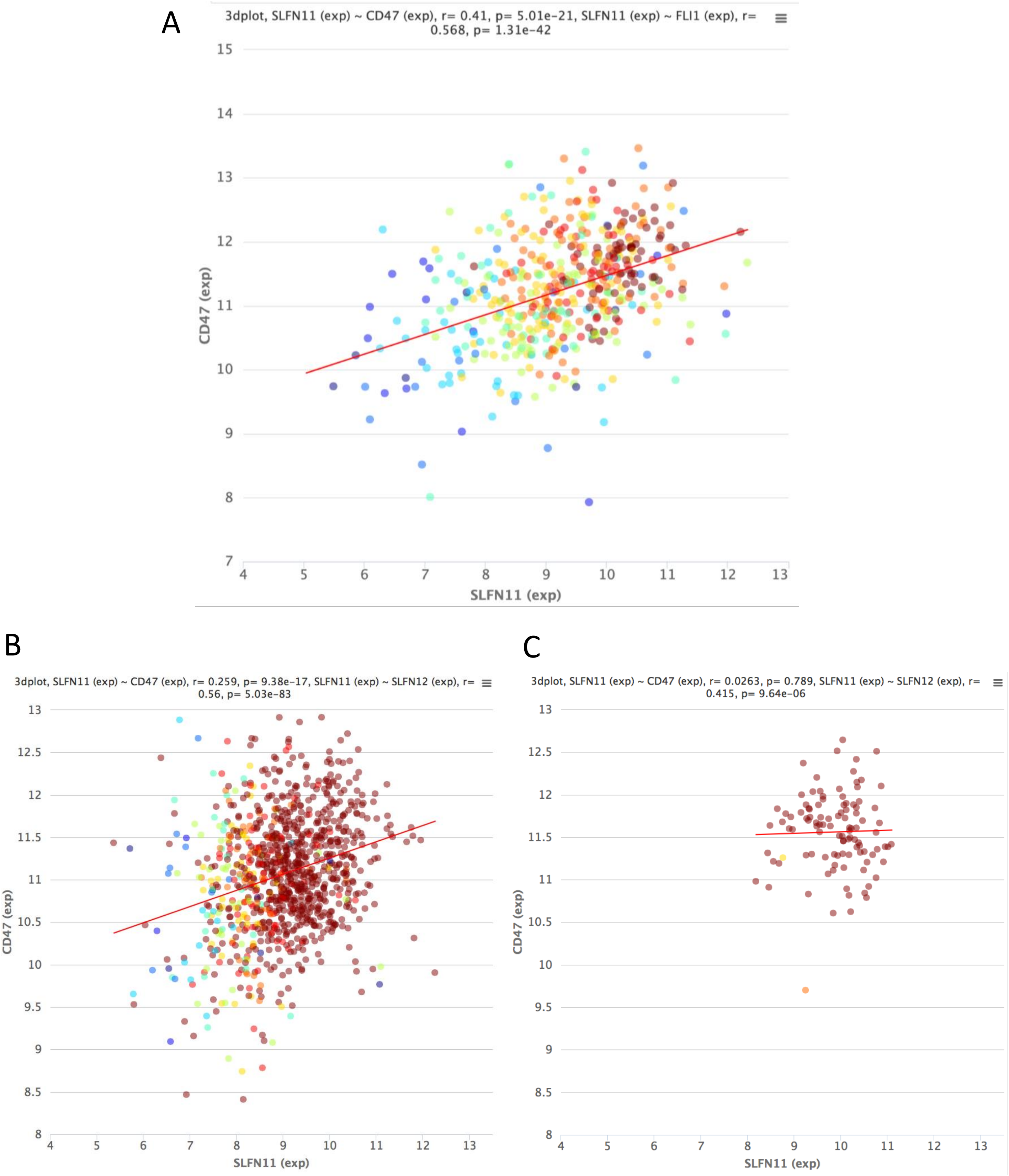

Supplementary Figure 2

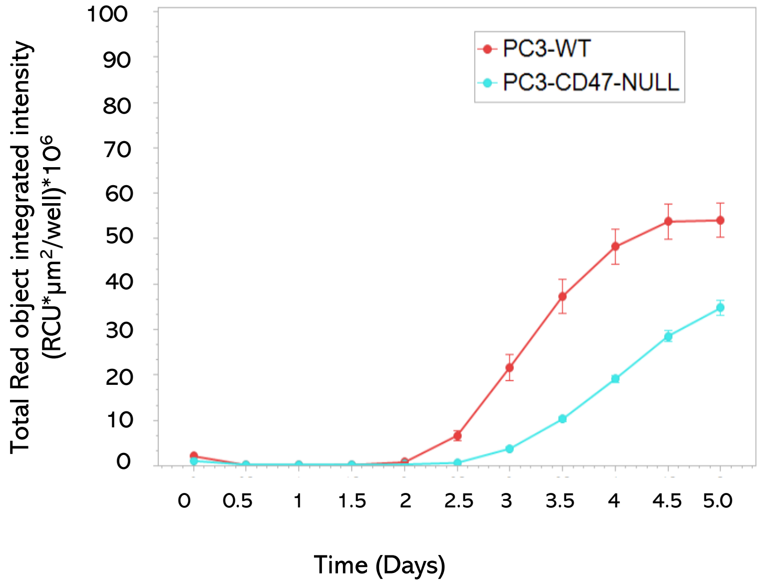

Supplementary Figure 3

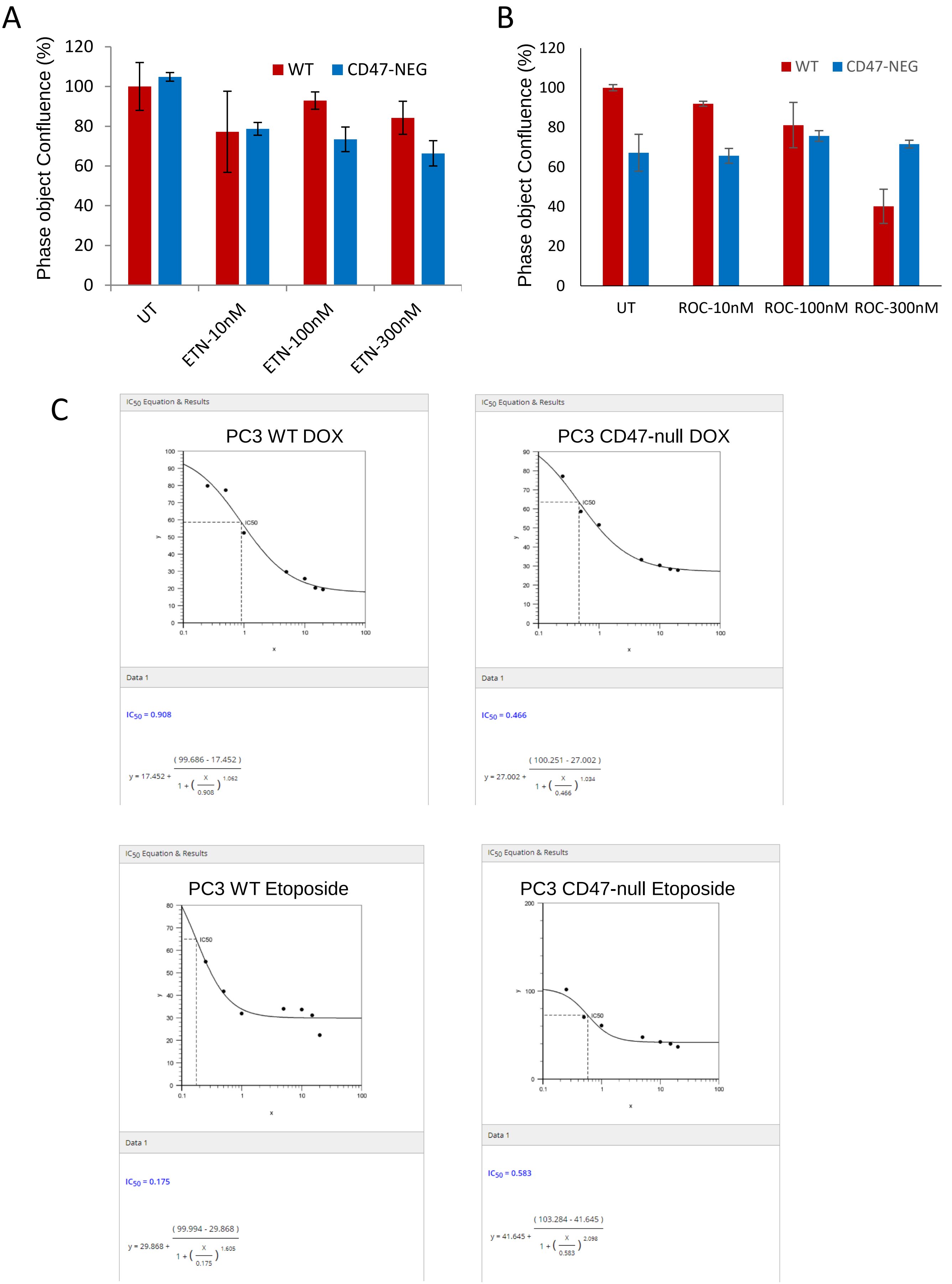

Supplementary Figure 4

Breast carcinoma (CD47/SLFN11 coexpression r = 0.245)

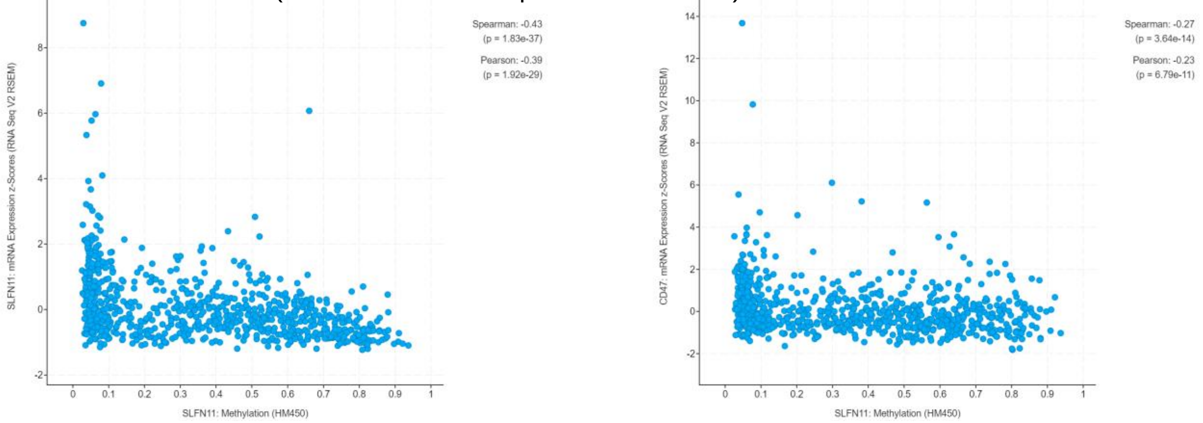

Cutaneous melanoma (CD47/SLFN11 coexpression r = 0.338)

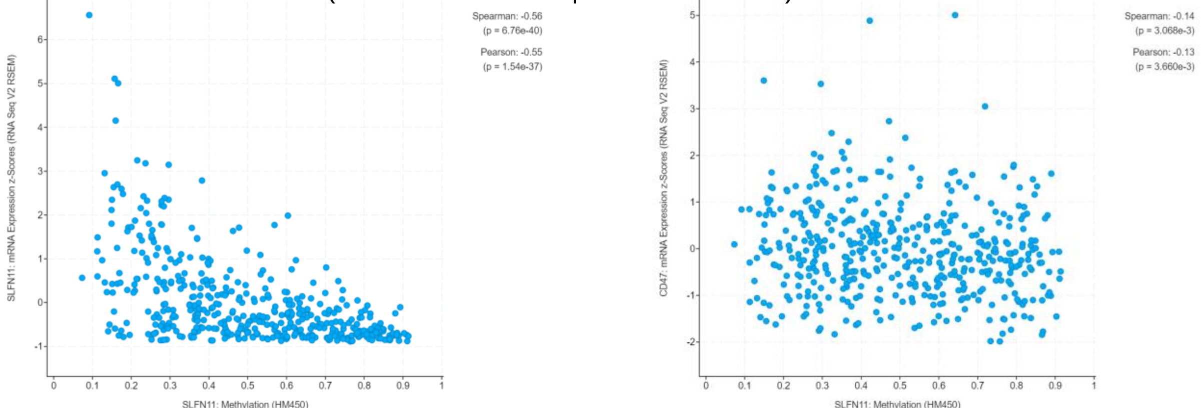

Colorectal adenocarcinoma (CD47/SLFN11 coexpression r = 0.046)

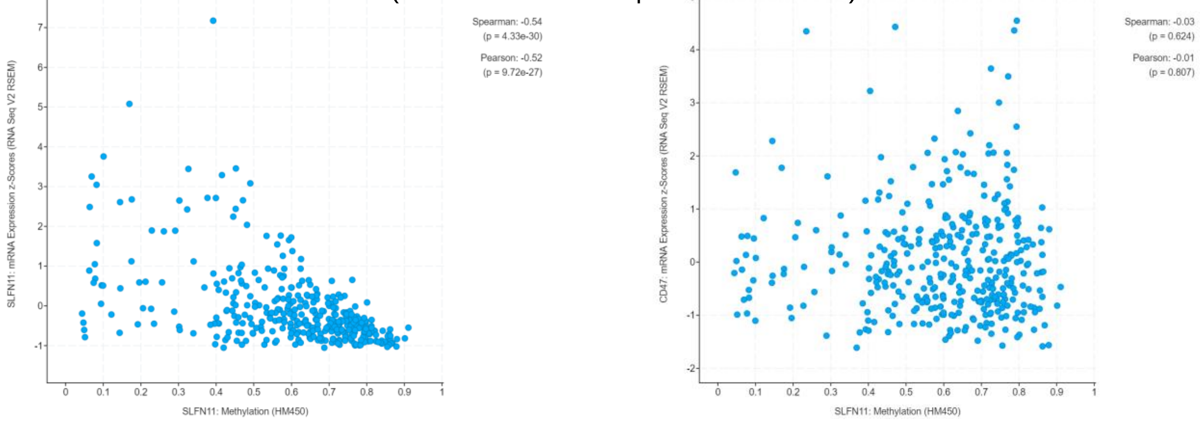

Soft tissue sarcoma (CD47/SLFN11 coexpression r = 0.215)

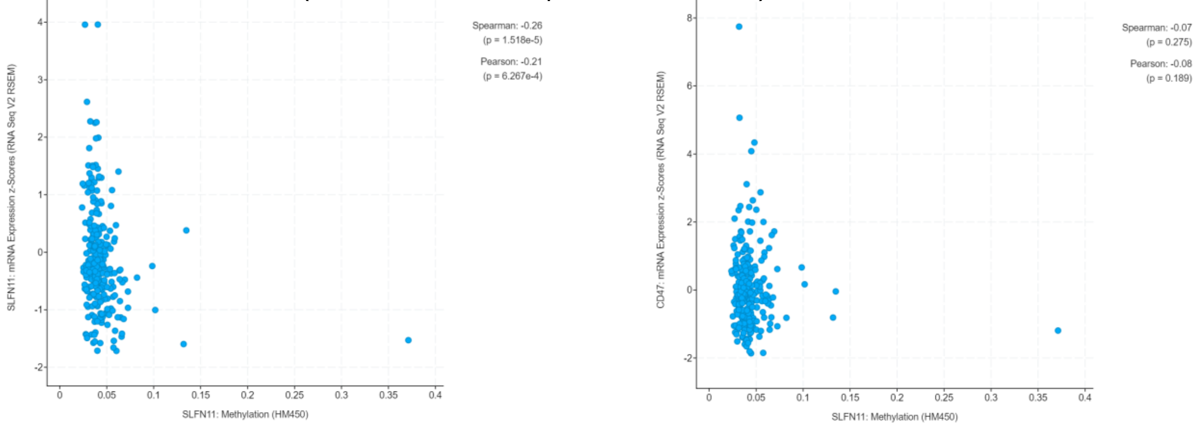

Supplementary Figure 5

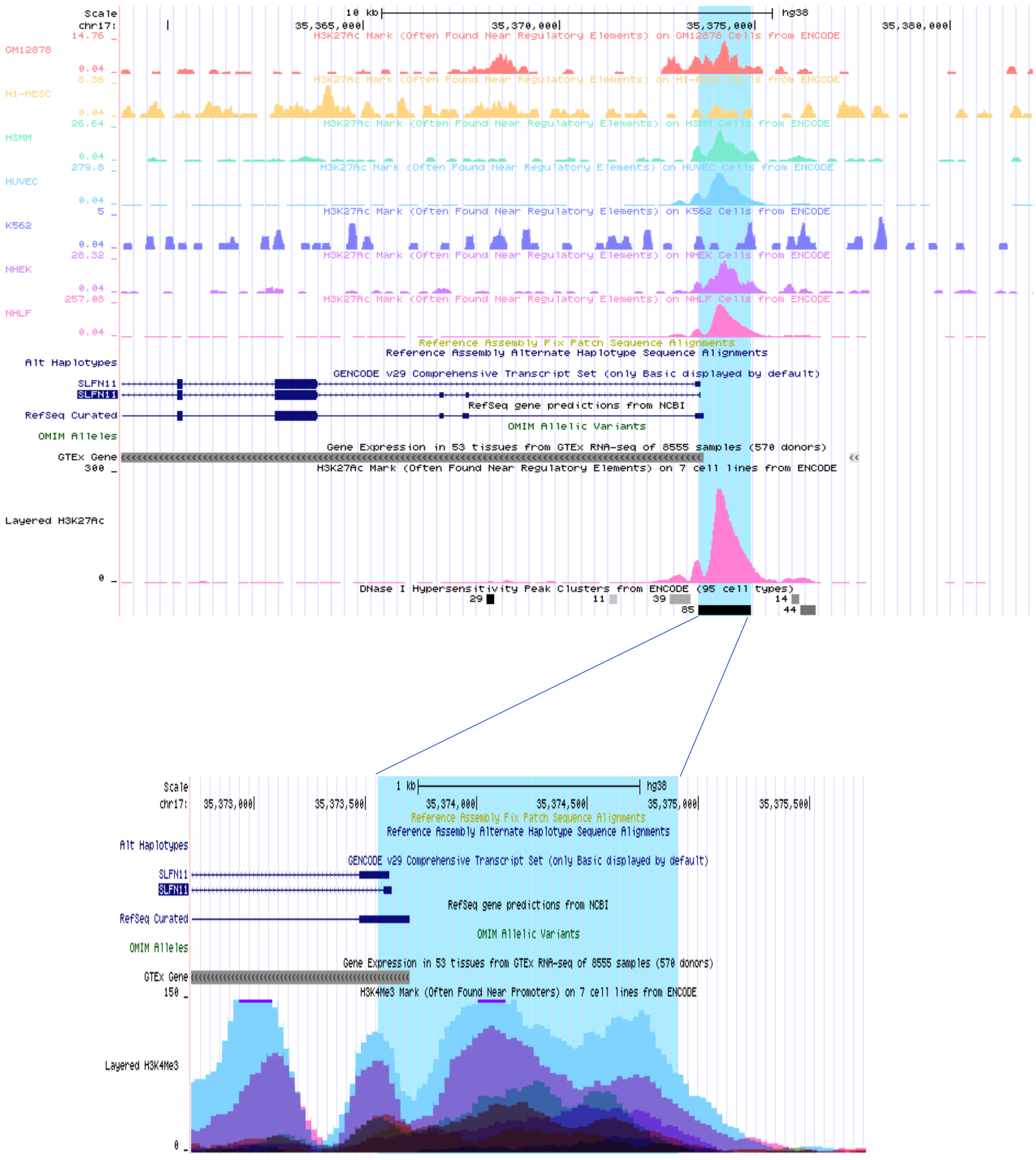

Supplement: Supplementary file 1 [file Data_Sheet_1.PDF]
